# *Plasmodium* parasite as an effective hepatocellular carcinoma antigen glypican-3 delivery vector

**DOI:** 10.18632/oncotarget.15806

**Published:** 2017-03-01

**Authors:** Quan Liu, Yijun Yang, Xuefang Tan, Zhu Tao, Dickson Adah, Songlin Yu, Junnan Lu, Siting Zhao, Limei Qin, Li Qin, Xiaoping Chen

**Affiliations:** ^1^ Laboratory of Pathogen Biology, State Key Laboratory of Respiratory Diseases, Center for Infection and Immunity, Guangzhou Institutes of Biomedicine and Health (GIBH), Chinese Academy of Sciences, Guangzhou, China

**Keywords:** GPC3, vector, plasmodium parasite, hepatocellular carcinoma, immunotherapy

## Abstract

We have previously demonstrated that malaria parasite infection has an anti-tumor effect in a mouse model. This research aimed to investigate the possibility of using *Plasmodium* parasite as a novel vaccine vector for hepatocellular carcinoma (HCC) immunotherapy. We constructed a *Plasmodium* yoelii 17XNL strain (P.y) expressing murine glypican-3 (GPC3) protein (*P*.*y*-GPC3), and examined its therapeutic potency in a murine Hepa1-6-induced hepatoma model that highly expressed GPC3 protein. The prerequisites for invoking a CD8+ T cell response were assessed after *P*.*y*-based immunization, which included obviously increased concentrations of T helper cell type 1 (Th1)-associated cytokines, such as IL-2, IFN-γ and TNF-α, in serum and preferential expansion of the CD8α+ dendritic cell (DC) subset with higher expression of CD80 and CD86 molecules. Compared with uninfected and wild-type *P*.*y*-infected mice, a significant GPC3-specific cytotoxic T lymphocyte (CTL) response was detected in *P*.*y*-*GPC3* vaccinated mice. Furthermore, *P*.*y*-*GPC3*-based vaccination dramatically inhibited Hepa1-6-induced tumor growth in the implanted HCC and prolonged the survival of tumor-bearing mice. We concluded that a *Plasmodium*-based vector is highly efficient in inducing tumor antigen-specific T cell-mediated immunity and protection against tumor cells. More broadly, this strategy supported our hypothesis that *Plasmodium* parasites, as novel therapeutic antigen vectors, may be applicable to tumor immunotherapy for patients with HCC.

## INTRODUCTION

Hepatocellular carcinoma (HCC), which accounts for 85–90% of primary liver cancers, is the third most common causes of cancer mortality worldwide [1, 2]. Estimated 782,500 new liver cancer cases and 745,500 deaths occurred in 2012 [3]. The traditional treatments, including surgery, chemoembolization and chemotherapy, are often ineffective in controlling HCC, and up to 50% of patients are not good candidates for these conventional treatments [4, 5]. Immunotherapy has recently become a refined strategy for tumor treatment [6–10], and a tumor vaccine is an important option for patients with HCC [11, 12]. In a phase I/II trial of alpha fetal protein (AFP) peptide-pulsed DCs or GPC3-derived peptide vaccine, a transient CD8+ T cell response was detected in patients with HCC and overall survival (OS) was positively associated with the specific cytotoxic T lymphocyte (CTL) response [13, 14]. More encouraging outcomes were obtained in other HCC preclinical and clinical research [15–17]. However, many vaccines induced effective but not prolonged T cell mediated immunity. An appropriate delivery vector may solve the problems of antigen retention and release *in vivo* and generate robust and long-lasting forms of immune responses, particularly in specific CTLs, which are critical for immunological control of HCC [12].

Some studies have shown that pathogenic infections in the host have the ability to suppress tumor growth [18–22]. *Plasmodium* parasite, which is an intracellular protozoan, is the most common parasitic agent in humans and animals. We have previously demonstrated that infection with malaria parasites has an anti-tumor effect through induction of innate and adaptive immunity in a murine Lewis lung cancer model [23]. *Plasmodium* parasites have components such as glycosylphosphatidylinositol (GPI) anchors [24], genomic DNA, haemozoin [25] and others that present as pathogen-associated molecular patterns (PAMPs), which are rapidly recognized by the innate immune system. Consequently, activation of macrophages, DCs, natural killer (NK) cells, γδ T cells, natural killer T cells (NKT), CD4+ T cells and CD8+ T cells occurs during its blood-stage infection [26]. It is supposed that host immunity invoked by the *Plasmodium* parasite infection may enhance anti-tumor immunity. In addition, we propose that the *Plasmodium* parasite could be an HCC vaccine vector for more rationales: (i) foreign protein can be expressed in parasites over a long period of infection. (ii) certain critical immunostimulatory characteristics of the parasites can enhance CTL-mediated anti-tumor immunity and prolong immune responses, and (iii) *Plasmodium* parasites are potent triggers of antigen-specific cytotoxic activity in CD8+ T cells.

Significant progress has been made in identifying members of HCC-associated antigens as described previously [12]. GPC3 is one of the well-studied HCC-associated tumor antigens, and its specific CTL has been identified both in patients with HCC and in mice [27–29]. The results of phase I/II trial showed that a GPC3-derived peptide vaccination was well-tolerated, and that OS was significantly longer in patients with high GPC3-specific CTL frequencies than in those with low frequencies of CTL [14]. The GPC3 peptide vaccine, as an adjuvant therapy, improved the 1-year survival rate in GPC3-positive HCC patients who had received radiofrequency ablation (RFA) therapy or surgery [30]. Furthermore, GPC3-targeted chimeric antigen receptor (CAR) T cells have recently been explored and achieved a great therapeutic effect [31]. In short, GPC3, a carcinoembryonic antigen, is an ideal tumor antigen for HCC immunotherapy with its special expression in HCC and highly immunological properties [32].

In this study, we chose *Plasmodium yoelii* 17XNL, a murine *Plasmodium* strain, as an HCC cancer vaccine vector capable of expressing GPC3 protein. The anti-tumor effect and immunological mechanisms of the *Plasmodium* were examined in a murine HCC model using C57BL/6 mice. The modified *Plasmodium* parasite induced host innate immunity and generated a GPC3-specific T cell response *in vivo*, resulting in inhibited HCC growth and prolonged mouse survival. In conclusion, the *Plasmodium* parasite might be a good candidate for a therapeutic HCC vaccine.

## RESULTS

### GPC3 protein is highly expressed in Hepa1-6 cells and Hepa1-6-induced HCC tissues in mice

**Figure 1:**
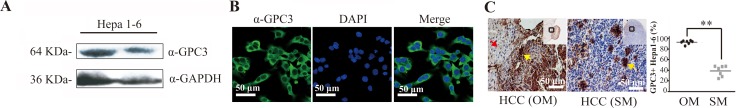
GPC3 protein is highly expressed in Hepa1-6 cells and Hepa1-6 cell-induced HCC tissues in mice (**A**) Western blot analysis of GPC3 protein in Hepa1-6 cells. (**B**) Localization of GPC3 in Hepa1-6 cells using confocal microscopy. (**C**) Immunohistochemical analysis of GPC3 in the HCC tissues. The above data is a representation of four repeated experiments. Scatter plot show the mean percentage ± SD of GPC3 positive Hepa1-6 cells. Statistical differences between groups are indicated by the *P* values (*P ≤ 0.05, **P ≤ 0.01, ***P ≤ 0.001) and each symbol stands for the percentage of intended cells in an individual microscopic field. SM: subcutaneous model, OM: orthotopical model. Bars: 50 μm.

First, we confirmed that Hepa1-6 cells expressed glypican-3 (GPC3) protein at the predicted size (Figure 1A) that was primarily located in the cell cytoplasm (Figure 1B). Furthermore, we used Hepa1-6 cells to establish HCC models and performed an immunohistochemical analysis of GPC3 in the HCC tissues. As shown in Figure 1C, GPC3 protein was expressed in both subcutaneously and orthotopically implanted HCC tissues (areas marked by a yellow arrow), but the normal liver tissues did not express GPC3 protein (areas marked by a red arrow). Compared with subcutaneously implanted HCC tissues, orthotopically implanted HCC tissues had a significantly higher expression of GPC3 protein (*P* = 0.002), and the percentage of Hepa1-6 cells that expressed GPC3 protein was more than 90%. These two HCC mouse models could be used in a cancer vaccine test.

### GPC3 protein is stably expressed in transgenic *Plasmodium* parasites

**Figure 2:**
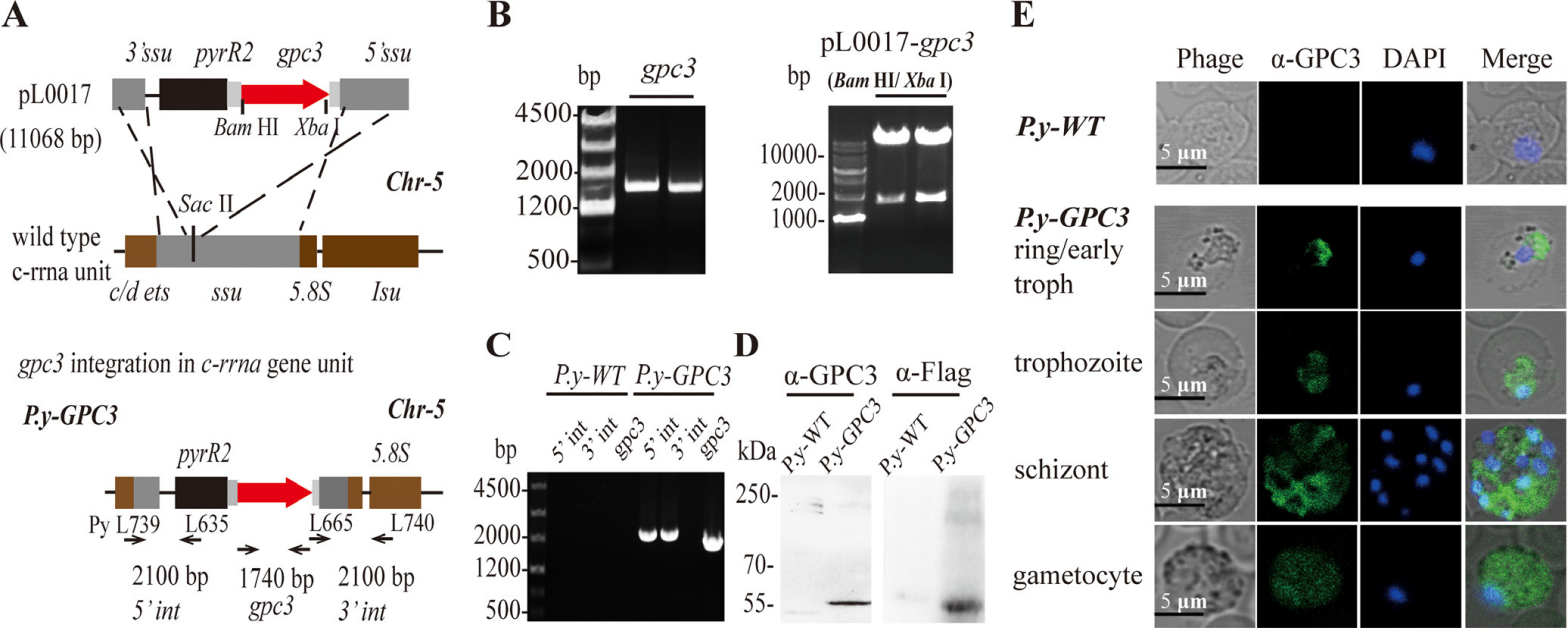
Expression of ectopic GPC3 by stably transfected P. *yoelii* 17XNL (**A**) Schematic representation of the pL0017-*gpc3* vector integrated into the *c-rrna* unit. (**B**) Gel electrophoresis and DNA analysis of the *gpc3* cloned from cDNAs of Hepa1-6 cells (left) by PCR, and identification of the pL0017-*gpc3* vector (right) by restriction enzyme analysis (*Bam* HI/*Xba* I). (**C**) Correct integration of the vector in *P.y*-GPC3. PCR was performed with genomic DNA from *P.y-GPC3* and *P.y-WT* (negative control). The 5′ integration site (*5′ int*, primers PyL739/L635) and 3′ integration site (*3′ int*, primers L665/PyL740) were verified, as well as the *gpc3* gene. (**D**) Detection of GPC3 expression in *P.y-GPC3* by western blot using anti-GPC3 (rabbit) or anti-Flag tag antibodies. (**E**) GPC3 expression by the four different stages of the *P.y-GPC3*. Parasitized erythrocytes were analyzed by immunofluorescence and *P.y-WT infected* erythrocytes were used for the negative control. Parasites stained with anti-GPC3 antibody (green), or DAPI for DNA, were visualized by confocal microscopy. Bars: 5 μm.

We engineered the wild-type of *Plasmodium yoelii* 17XNL (*P.y-WT*) to express the murine GPC3 protein with double Flag tags (*P.y-GPC*3) and its construction is described in Figure 2A. The *gpc3* gene was cloned and pL0017-*gpc3* plasmid was constructed (Figure 2B). For the analysis of the genotype of the recombinant parasite, primers were synthesized as described previously [33]. The parasite genomic DNA was extracted and analyzed using a polymerase chain reaction (PCR). As shown in Figure 2C, the successful clones of the *5′int*, *3*′*int* and *gpc3* genes confirmed that these intended genes were efficiently inserted into the parasite genomic DNA. Western blot analysis indicated that *P.y-GPC*3 successfully expressed the GPC3 protein at the predicted size (Figure 2D). The inserted *gpc3* gene was under the control of the *P. berghei* elongation factor 1-α (EF-1α) which is active throughout the parasite life cycle [34]. Immunofluorescence results revealed that the GPC3 protein was expressed throughout the blood stage of *P.y-GPC3* (Figure 2E), which includes the ring, trophozoite, schizont and gametocyte phases.

### *Plasmodium* parasite immunization triggers a Th1-type response at the early stage of infection in HCC-bearing mice

**Figure 3:**
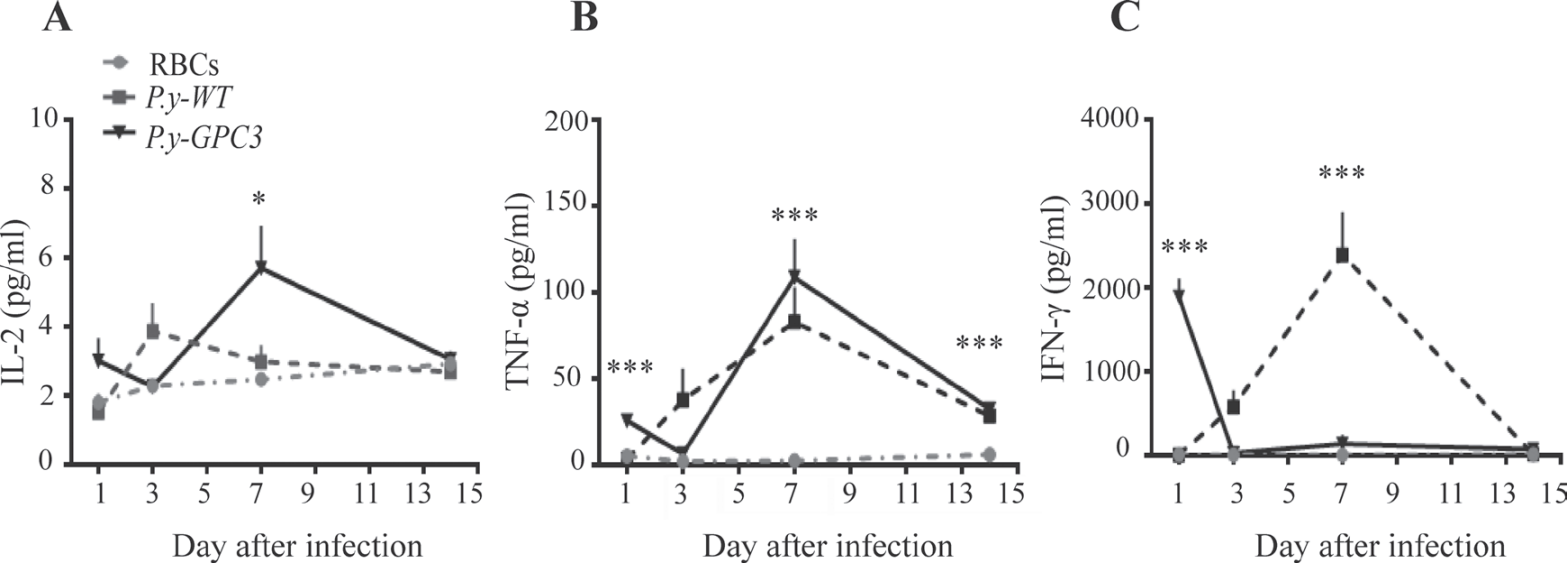
Detection of Th1-type cytokines in serum during early parasite infection Concentrations of IL-2 (**A**), TNF-α (**B**) and IFN-γ (**C**) in serum were determined by FACS with a multi-analyte flow assay kit. Serum was acquired from three different mouse groups (RBCs, *P.y-WT* and *P.y-GPC3* treated mice, *n* = 5/group) at four different time points. The values shown represent the mean and standard errors of the means. Statistical differences between groups are indicated by the *P* values (*P ≤ 0.05, **P ≤ 0.01, ***P ≤ 0.001). The above data is a representation of four repeated experiments.

To assess the anti-tumor potentiality of the modified *Plasmodium*-based vaccine, we checked its immunostimulatory effect on T helper (Th) cells in HCC-bearing mice. Th1-related cytokines in mouse serum were analyzed. The results showed that infection with the malaria parasite significantly activated a Th1-type response. Concentrations of IL-2, IFN-γ, and TNF-α rapidly increased in the early stage of infection and reached to a peak within the first week (Figure 3). On the 7th day, *P.y-GPC3* vaccination invoked high expression of IL-2 in serum, compared to the other two groups (both *P* ≤ 0.05, Figure 3A). TNF-α expression levels were high during the *P.y-GPC3* infection on the 1st, 7th or 14th different days and were significantly different from those of uninfected mice (all *P* ≤ 0.01). At the same time, TNF-α also had a high expression in *P.y-WT* infected mice (Figure 3B). Surprisingly, unlike its peak happened in the 7th day of *P.y-WT* infection, IFN-γ had a peak in the first day of *P.y-GPC3* infection (both *P* ≤ 0.01, Figure 3C). These results confirmed that malarial infection had a function in activation of a Th1-type response in *Plasmodium*-based vaccinated mice.

### Vaccination with *P.y-GPC3* induces expansion of the CD8α+ DC subset, with preferential expression of CD80 and CD86 molecules

**Figure 4:**
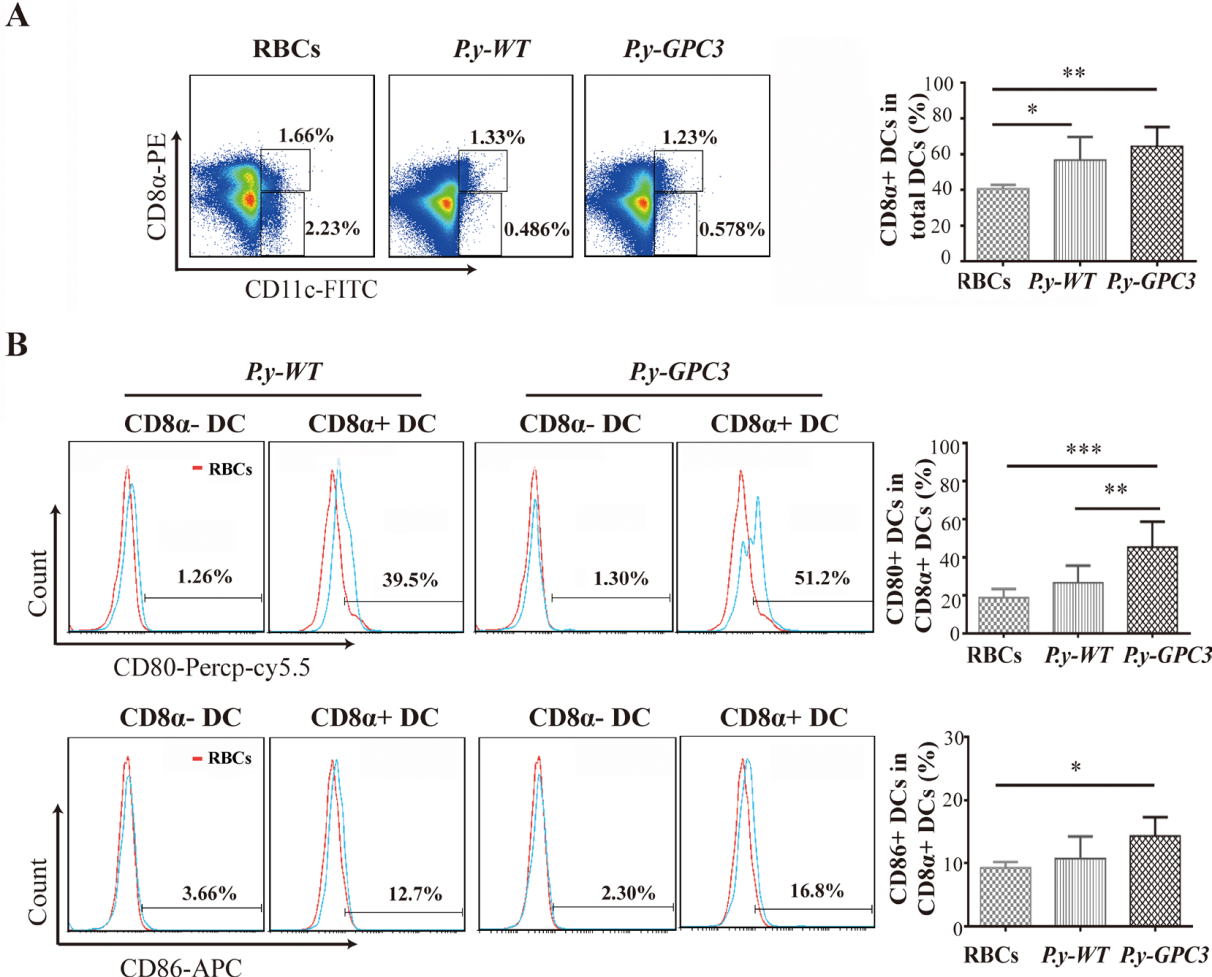
Expansion of CD8α+ DC subset with preferential expression of CD80 and CD86 molecules in *P.y-GPC3*-vaccinated mice (**A**) The percentage of CD8α+ DC or CD8α- DC population in total splenocytes (left) and the percentage of CD8α+ DC population in total CD11c+ DCs (right). Splenocytes were measured from three different mouse groups (RBCs, *P.y-WT* and *P.y-GPC3* treated mice, *n* = 5/group). (**B**) The percentages of CD80 and CD86 positive DCs either in CD11c+ CD8α- DCs (CD8α- DC) or CD11c+ CD8α+ DCs (CD8α+ DC). The CD80/CD86 expression in the *P.y-WT* and *P.y-GPC3* treated mice (blue lines) and their expression in the uninfected group in the control (red lines) are shown (left). Furthermore, the percentages of CD80 and CD86 positive DCs in CD8α+ DC were measured in each histogram, and the differences between these three groups are indicated by the *P* values (*P ≤ 0.05, **P ≤ 0.01, ***P ≤ 0.001). All histograms were based on 10,000 cells satisfying a positive gate set of CD11c-FITC. Bar graphs show the mean percentage ± SD of intended positive DCs. FACS data shown are representative of four independent experiments.

Dendritic cells (DCs) are a group of heterogeneous myeloid cells, and studies have shown that CD8α+ DCs have the ability to direct the differentiation of Th1-type cells and present antigens to CD8+ T cells, which is important in activating immune responses against tumors in vaccination strategies [35–38]. We analyzed the effect of immunization with *Plasmodium* parasite on DC polarization and maturation in a mouse HCC model. Spleens were isolated from mice on the fourteenth day after vaccination, and the percentage of CD8α+ DCs within the total splenic CD11c+ DCs population was determined. As displayed in Figure 4A, infection with *P.y-WT* or *P.y-GPC3* led to a decrease in percentage of splenic CD8α- CD11C+ DCs compared with uninfected mice (0.486% or 0.578%, vs 2.23%). In the total splenic CD11c+ DC population, *P.y-WT* or *P.y-GPC3* parasite-infected HCC-bearing mice activated a higher percentage of CD8α+ DCs than their control mice counterparts (56.67% or 64.43%, vs 40.51%), and the difference was statistically significant (*P* = 0.027 and *P* = 0.002).

Because the function of DCs in modulating immune responses depends largely on their expression of co-stimulatory molecules, we further analyzed DC surface markers, such as CD80 and CD86. As shown in Figure 4B, compared with the CD8α- DCs in the spleen of *P.y-WT*-infected mice, the CD8α+ DCs expressed increased levels of CD80 (1.26% vs 39.5%), and CD86 (3.66% vs 12.7%). The similar results were obtained in *P.y-GPC3*-infected mice (CD80: 1.3% vs 51.2% and CD86: 2.3% vs 16.8%). This result indicated that CD8α+ DCs were more mature DCs than CD8α- DCs. Exogenous GPC3 expression in parasites did not decrease the percentage of the CD8α+ DC subpopulation and its co-stimulatory molecules in mice. Furthermore, we found that *P.y-GPC3-*vaccinated mice had a higher expression of CD80 (both *P* = 0.01) and CD86 molecules (*P* = 0.013) in CD8α+ DCs than control mice and even *P.y-WT*-vaccinated mice (Figure 4B). The maturation of CD8α+ DCs provided prerequisites for the induction of tumor antigen-specific CD8+ cytotoxic T cells.

### Antigen-specific CD8+ cytotoxic T cell response is generated during the blood-stage infection

**Figure 5:**
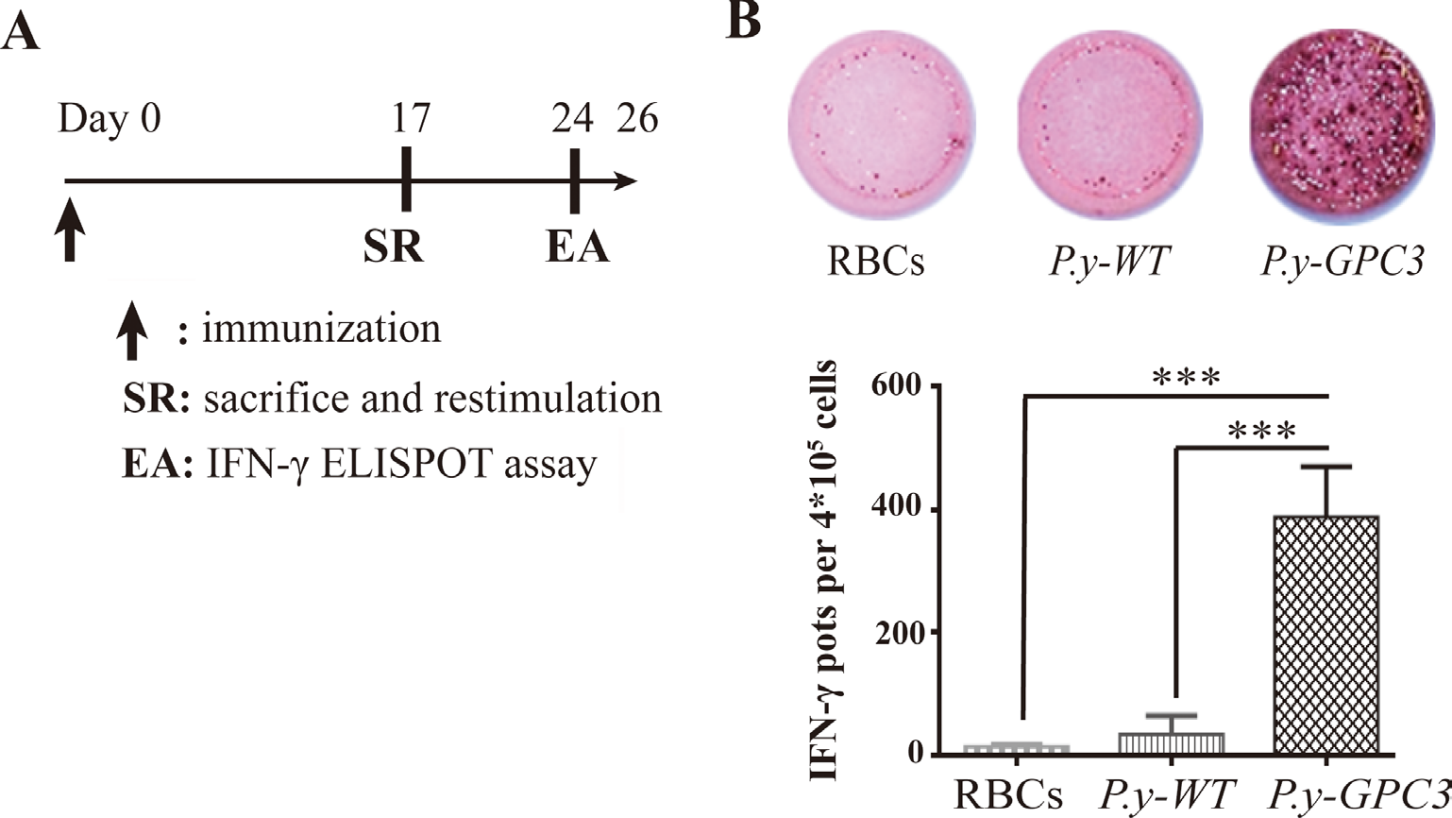
Identification of cytotoxic T cell responses in mice with *P.y-GPC3* immunization (**A**) Schedule of *Plasmodium*-based vaccination in three groups of mice (RBCs, *P.y-WT* and *P.y-GPC3* treated mice, *n* = 4/group). (**B**) Identification of GPC3-specific CTLs by IFN-γ ELISPOT assays using purified CD8+ T, and BM-DCs pulsed with GPC3 protein as target cells (***P ≤ 0.001). Representative data are shown. The data shown are the mean SD of IFN-γ pots per 4 × 10^5^ CD8+ T cells for four mice per group. ELISPOT assays were performed in triplicate and these data are representative of four independent experiments.

Cytotoxic T lymphocytes (CTLs) are a critical component of the immune responses to tumors. We followed a vaccination schedule (Figure 5A) for the mice to check whether infection with *P.y-GPC3* induced a GPC3-specific CTL response. At day 17, CD8+ T cells were enriched from splenocytes and the purity reached to 94.7% (data not shown). The parasite immunogenic potential to GPC3-specific cytotoxic T lymphocytes was measured by Interferon-γ Enzyme-linked Immunospot (ELISPOT) assays (Figure 5A). The results showed that *P.y-GPC3* significantly induced high IFN-γ expression in CD8α+ T cells (P≤0.001, Figure 5B) and it indicated that these IFN-γ-positive expression cells were GPC3-stimulated CD8α+ T cells. We did not detect IFN-γ expression in CD8+ T cells from the *P.y-WT* infected and uninfected mice. These findings indicated that, compared with the wild type and no parasite-infected mice, the GPC3-expressing *Plasmodium* parasite had the capacity to induce GPC3-specific CTLs *in vivo*.

### Infection with genetically modified parasites in mice suppresses HCC growth and improves survival

**Figure 6:**
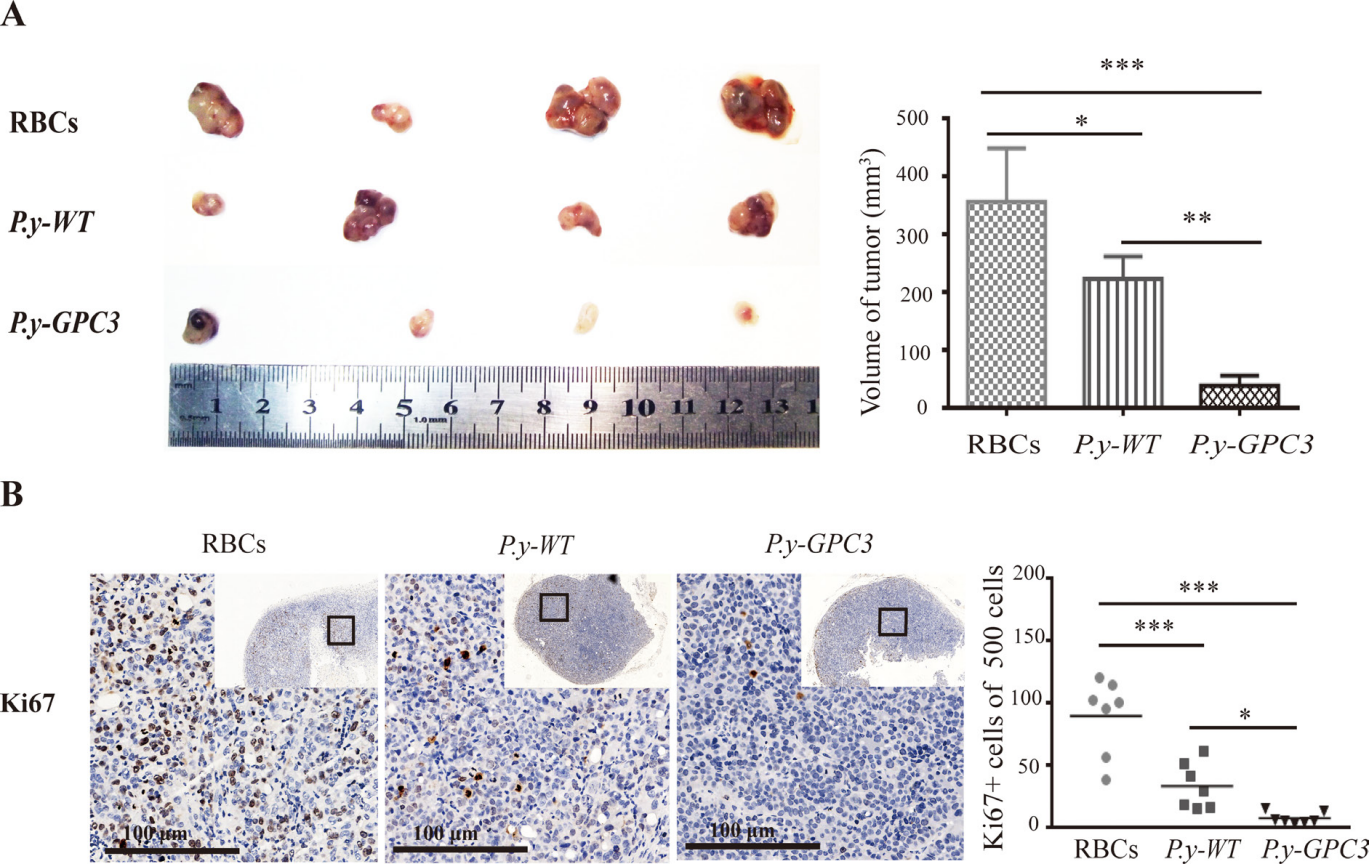
Suppression of the HCC growth after *Plasmodium*-based immunization (**A**) Measurement of tumor volumes at fourteen days after different immunization (RBCs, *P.y-WT* and *P.y-GPC3* treated mice, *n* = 4/group). (**B**) Immunohistochemical staining (left) and assay quantification (right) for Ki-67 in different treated HCC tumors. Each symbol stands for the percentage of intended cells in an individual microscopic field in the scatter graph. Bar graphs show the mean percentage ± SD. Statistical differences between groups are indicated by the *P* values. *P ≤ 0.05, **P ≤ 0.01, ***P ≤ 0.001. Bars: 100 μm. Data are representative of four independent experiments.

The effect of *Plasmodium*-based vaccination on the growth of HCC in mice was evaluated. Following the schedule described in Figure 5A, we measured tumor volumes and detected the Ki-67 expression in tumor tissues with immunohistochemical analysis. The growth of tumor cells was clearly suppressed in the *P.y-GPC3* infected mice group compared to those tumors in the *P.y-WT* (*P* < 0.05) and the control mice group (P≤0.001, Figure 6A). The wild type of *Plasmodium* parasite also had anti-tumor effect on Hepa1-6 induced HCC model compared to those in the control group (*P* < 0.05). The Ki67 expression in the tumor was also dramatically inhibited in both of *P.y-GPC3* and *P.y-WT* vaccinated mice (*P* ≤ 0.001, Figure 6B).

**Figure 7:**
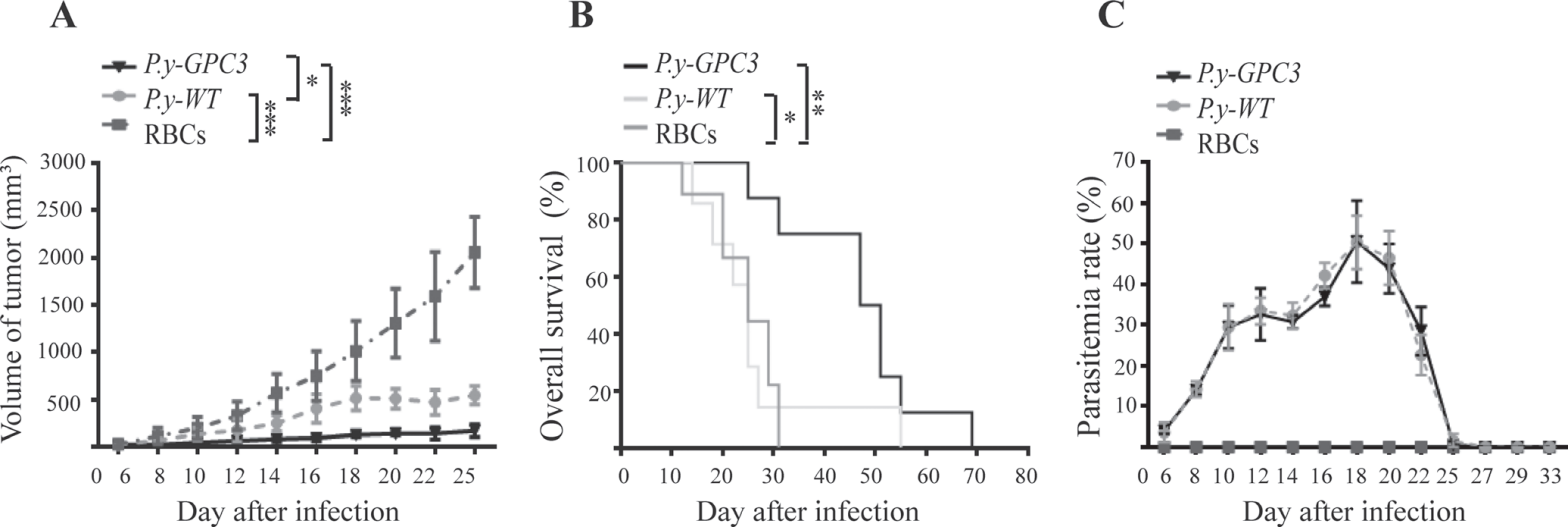
Infection with *P.y-GPC3* inhibited mouse HCC growth and prolonged survival life for an extended period Three groups (RBCs, *P.y-WT* and *P.y-GPC3*) of mice were immunized as described in the Methods. Tumor volumes were measured over time (**A**). We determined the survival end point at approximately 70 days and recorded mouse survival (**B**) and the parasitaemia rate in mice (**C**). Graphs shows means ± SD. Statistical differences between groups are indicated by the *P* values and a log-rank test (*P ≤ 0.05, **P ≤ 0.01, ***P ≤ 0.001). Error bars represent standard error of the mean. These data are representative of four independent experiments.

In addition, we performed an extended observation of tumor-bearing mice that received a subcutaneous (s.c.) injection of different *Plasmodium*-based vaccinations. On the 10th day after vaccination, we checked the differences in the tumor sizes of *P.y-GPC3*-infected mice and control mice. After 25 days of infection, the growth of the tumors was still clearly suppressed in the *P.y-GPC3*-infected mice compared to the *P.y-WT* (*P* < 0.05) and control mice (*P* < 0.001, Figure 7A). More importantly, *P.y-GPC3*-infected mice received good protection and experienced prolonged survival time (Figure 7B). The results showed that GPC3 expression also did not inhibit the parasite growth (Figure 7C).

## DISCUSSION

A successful antigen-based tumor vaccine for HCC depends largely on an appropriate type of delivery vector. Many traditional vaccine vectors are used intensively [39]. However, many of them induce effective but not prolonged T cell-mediated immunity. Recently, some of protozoan parasites have been studied as potential cancer delivery vectors, such as *Trypanosoma cruzi* [40] and *Toxoplasmosis gondii* [41]. Our previous studies demonstrated that *Plasmodium* parasite could be a delivery vector for a conceptual human immunodeficiency virus (HIV) vaccine [42] and its immunization had an anti-tumor effect in a mouse model [23]. Here, we use it as a delivery vector for an HCC-associated vaccine. The host's stimulated immune mechanisms induced by the modified *Plasmodium* infection are considered and exploited to inhibit tumor growth.

Our study showed that both *P.y-WT* and *P.y-GPC3* infection increased high levels of proinflammatory cytokines, such as IL-2, TNF-α and IFN-γ, which leads to activation of defense response effectors against tumor cells [43]. This result corresponded with previous researches on *Plasmodium* infection where malaria is characterized by outstanding cytokinaemia, and protective immunity is mediated by a Th1-type response and IFN-γ [44]. Meanwhile, we detected no significant concentration changes in Th2 associated cytokines in the model, which included IL-4, IL-5 and IL-13 (data not shown).

Except the polarization of Th1 responses [45], maturation of DCs, in particular of the CD8α+ DCs [46], is also positive to CTL-mediated anti-tumor immunity [47, 48]. Fourteen-day time point was selected for measuring the immunological parameters because it was within the period of peak parasitaemia and possible highest GPC3 expression level. DCs derived from *P.y-WT* or *P.y-GPC3* infected mice had a significantly higher percentage of CD8α+ DCs than those in uninfected mice. Similar results were observed in the expression of CD80 abd CD86 molecules in CD8α+ DCs, compared to their counterpart CD8α- DCs. It suggested that a preferential expansion of the CD8α+ DCs in total CD11c+ DCs occured after the *Plasmodium* immunization. It also revealed that a *Plasmodium*-based vector could effectively establish the prerequisites for triggering antigen-specific CTL responses.

It is well studied that the liver and blood stages of *Plasmodium* infection involve the CD8+ T cell response, which participates in the host protection against parasites [49–51]. Utilizing *Plasmodium* parasites as a delivery vector, ectopic tumor antigens can be successfully expressed at its blood stage. The parasitemia reaches to near 50% in 2–3 weeks after infection and then self-cures after another 1–2 weeks. This provides an extended period due to antigen retention and slow release in parasites, which is ideal for induction of protective immunity. We found that GPC3 protein expressed in parasites could be presented and initiated GPC3-specific CTLs. Although studies showed that no MHC class I molecules are displayed on mature erythrocytes, parasite infected erythroblasts collaborated with phagocytes may be required for induction of the MHC class I-restricted CD8+ T cell responses in mice [51]. *P. vivax* and *P. falciparum* were also proved to preferentially invade reticulocytes [52–54]. Meanwhile, our previous study indicated that a murine parasite which preferentially infects mature erythrocytes, *P. berghei* expressing HIV-1 Gag protein could induce strong Gag-specific CD8+ CTL responses in vaccinated mice [42]. This suggested that immature infected erythrocytes may be unnecessary for inducing CD8+ CTL responses. Importantly, other researchers have shown that blood-stage *Plasmodium* infection induces CD8+ T lymphocytes to parasite-expressed antigens and it is largely regulated by CD8α+ DCs [50]. Thus, the prolonged antigen stimulation and preferential induction of CD8+ T cell responses seen in mice with *P. yoelii* 17XNL could be promising to duplicate in humans.

In this study, the initial hypothesis was that *Plasmodium* parasites first activated the CD4+ Th1-type response, and then triggered a strong and long-lasting GPC3-specific CTL *in vivo*. Vaccination with *P.y-GPC3* led to the activation of immunological mechanisms in killing or controlling the growth of tumor cells. In fact, *Py-GPC3* could suppress the tumor growth but could not eradicate tumor cells, which might be due to small portion of GPC3-expressing tumor cells in subcutaneous HCC tissues. Thus, extension of this current work could further utilize the orthotropic model. In addition, the wild type of malaria parasite also has some anti-tumor effects, and its mechanism is more associated with the stimulated immune responses induced by parasites and others, such as the inhibition of tumor angiogenesis with *Plasmodium*-derived exosomes (unpublished data). In conclusion, we demonstrated a novel HCC immunotherapy that utilized *Plasmodium* parasites as delivery vectors to express tumor-associated antigens. The immunized mice received protective immunity against HCC growth and experienced a prolonged survival time. Lastly, the ethical consideration of using *Plasmodium* parasites to treat patients with HCC has been envisaged. The use of *Plasmodium* parasites as live vectors for an HCC vaccine should be further explored. For instance, the parasites at least need to be attenuated or attenuated *Plasmodium* sporozoites [55] could be tried as live vectors.

## MATERIALS AND METHODS

### Mice, parasite and cells

6-8 weeks old female C57BL/6 (B6) mice were purchased from *Shanghai Slack Laboratory Animal Co., LTD* (license: SCXK (HU) 2007-0003, China). Mice were maintained under the institutional guidelines of the Animal Center of the Guangzhou Institutes of Biomedicine and Health. Mice were housed in specific pathogen-free (SPF) conditions, with a 12-hours light cycle and food and water at *ad libitum*. All animal experiments were carried out with the standard guidelines for the care of animals, which were approved by the Welfare Committee of the Center of Experimental Animals (Guangzhou, China). The nonlethal *Plasmodium yoelii* 17XNL strain and pL0017 plasmid were kindly provided by the Malaria Research and Reference Reagent Resource Center (MR4). The Hepa1-6 cell line, a murine liver cancer cell line derived from B6 mice, was obtained from the Chinese Academy of Sciences Cell Bank (Shanghai, China).

### Animal grouping and inoculation

Mice were randomized into three groups of fifteen (15) mice per group. Mice were injected (s.c.) with 5×10^5^ Hepa1-6 cells. The tumor cell-inoculated mice were then intraperitoneally injected with 5×10^5^ *P. yoelii* 17XNL parasitized erythrocytes (*P.y-WT*) or *P.y-GPC3* parasitized erythrocytes (*P.y-GPC3*). Mice injected with non-infected erythrocytes were used as the control group (RBCs). After fourteen (14) or seventeen (17) days post inoculation, mice were euthanized and spleens were harvested for further analysis.

### Determination of mouse parasitemia

Blood smears were obtained from tail veins, methanol-fixed, stained with 5% Giemsa solution (Sigma) and microscopically observed to determine parasitaemia in 1,000 erythrocytes. The percentage of infected erythrocytes was calculated as follows: Parasitaemia (%) = (number of infected erythrocytes×100)/total number of erythrocytes counted (1,000).

### Detection of GPC3 in Hepa1-6 cells and Hepa1-6 cell-induced HCC tissues in mice

Expression of GPC3 in Hepa1-6 cells was detected by western blot and fluorescence microscopy based on protocols reported in previous studies [56]. Cell nuclei were labeled with DAPI (4′, 6-diamidino-2-phenylindole, Sigma). The following antibodies were used in this section: GPC3 (middle region) antibody (Aviva Systems Biology Corp., San Diego, CA, rabbit, #ARP37665), anti-rabbit IgG-HRP antibody (Cell Signaling Technology, #7074); anti-GAPDH antibody (Thermo, mouse, #MA5-15738), anti-mouse IgG-HRP antibody (Cell Signaling Technology, #7076); GPC3 monoclonal (9C2) antibody (Thermo, mouse, #MA5-17083) and Alexa Fluor@488 donkey anti-mouse IgG (H+L) secondary antibody (Life Technologies, #1423052). Implanted HCC models were established with B6 mice (*n* = 4/group). Briefly, 5×10^5^ Hepa1-6 cells were injected subcutaneously (or orthotopically) into the right flank (or the liver) of the mice. Two weeks later, the mouse HCC tissues were dissected. Immunohistochemical detection of GPC3 was performed as described previously [57]. All cell cultures were free of mycoplasma and maintained in complete medium (RPMI 1640 or DMEM supplemented with 10% fetal bovine serum (FBS), 100 U/ml penicillin, and 100 μg/ml streptomycin).

### Modified parasite construction and characterization

RNA was extracted from Hepa1-6 cells using TRIzol Reagent (Invitrogen) according to the manufacturer's protocols. A PrimeScript^®^ II 1st strand cDNA Synthesis kit (Takara) was used to synthesize the cDNA, and then, *gpc3* was amplified using a Takara PCR amplification kit. The following primers were used: *gpc3*-*Bam* H I-forward: 5′-AGGATCCATGGCCGGGACCGTGCGCACC GCGT- 3′, *gpc3*-*Xba* I-2×Flag-reverse: 5′-GGTCTAGAGAGAC CTTACTTATCGTCGTCATCCTTGTAATCCTTATCGT CGTCATCCTTGTAATCGTGCACCAGGAAAAAAAA GCACGCC-3′. For homologous recombination, the *gpc3* gene was introduced into the genome of *P.y-WT* using a pL0017 plasmid by electroporation as previously described [58]. Pyrimethamine (Sigma) was used to select and clone pyrimethamine-resistant parasites in mice. Parasite genomic DNA was extracted using the DNeasy blood & tissue kit (QIAGEN). The integration of the exogenous gene into the parasite genome was confirmed by PCR analysis of parasite genomic DNA. GPC3 expression in parasites was detected with western blotting [42] and confocal microscopy (Leica) [59]. Monoclonal anti-Flag@M2 antibody (Sigma, mouse, #088K6018) was used for western blot and detailed data about other antibodies involved in this experiment were mentioned previously.

### Detection of Th1-type cytokines in serum

100-150 μl of blood was collected from the eye socket post inoculation, using capillary tubes, on the 1st, 3rd, 7th and 14th days. Serums were acquired after the coagulation of blood samples and stored at -80°C immediately. Th1-related cytokines in serum were determined by a multi-analyte flow assay kit (BioLegend) according to the manufacturer's protocols. Analyses were performed with a FACS Fortessa flow cytometer and the Legendplex software (BioLegend).

### Analyses of CD8α+ DC subset in HCC-bearing mice

On the 14th day after inoculation, mice were sacrificed and spleens and tumors were removed. Splenocytes were collected and stained with a combination of antibodies specific for CD11c-FITC, CD8α-PE, CD86-APC, CD80-PerCP-Cy5.5 (all purchased from eBioscience, San Diego, CA, USA). Splenocytes were then incubated in the dark at 4°C for 30 minutes and washed twice with FACS buffer (0.1% BSA and 0.05% sodium azide in PBS). Flow cytometry was performed using a BD FACS Arial flow cytometer and the results were analyzed with Flow-Jo software (Tree Star, Inc.).

### Generation of mouse bone marrow-derived DCs (BM-DCs)

BM-DCs (4×10^6^) from naïve B6 mice were generated as described [60] and cultured for 1 week in RPMI-1640 (GIBCO) supplemented with FBS (10%, GIBCO), 2-mercaptoethanol (2-ME, 50 μM, Sigma-Aldrich) and mouse granulocyte macrophage colony stimulating factor (GM-CSF, 20 ng/ml, PeproTech).

### Determination of GPC3-specific CD8α+ cytotoxic T cell response during parasite blood-stage infection

Spleens were isolated 17 days after inoculation and mashed using 70 μm filters. Splenocytes were harvested after depletion of RBCs by the hypotonic lysis buffer (BD Biosciences). CD8α-positive splenocytes were isolated by positive selection with anti-CD8α microbeads (Miltenyi Biotec) according to the manufacturer's protocol. The purity of CD8α+ T cells were analyzed with a combination of antibodies specific for CD8α-FITC and CD3e-PerCP-cy5.5 (all purchased from eBioscience, San Diego, CA, USA) by flow cytometry. Then, 2×10^6^ CD8α-positive splenocytes were co-cultured with prepared 5×10^5^ BM-DCs pulsed with 30 μg/ml murine GPC3 proteins (Sino Biological, Inc.) for restimulation. Seven days later, the suspensions containing 4×10^5^ CD8α+ T cells in T-cell medium were added to each well in ELISPOT 96-well plates and stimulated in triplicate with 30 μg/ml GPC3 protein at 37°C for 24 h. The detection of GPC3-specific T cells producing IFN-γ was performed using an ELISPOT kit (BD Biosciences) according to the manufacturer's protocol. Lastly, the plates were dried at normal temperature and the spots were counted with an ELISPOT Reader (CTL Limited) by capturing the images of individual wells. Spot-forming cells were defined as the average number of spots per 4×10^5^ CD8α+ T cells from triplicate wells.

### Measurement of tumor volume and mice survival

Tumors were measured 6 days post inoculation and the volumes were calculated from the formula, V = (*ab*^2^)/2, where *a* is the length of the tumor (mm), and *b* is the width of the tumor (mm). The survival end point of each mouse was determined by either spontaneous death or the presence of moribund signs. To ensure the welfare of the animals, the tumor size must not exceed 20 mm in any direction in each HCC-bearing mouse. If multiple tumors are presented, the combination of the two largest diameters may not exceed 20 mm for mice. When the tumor size exceeds 20 mm in any direction, mice were considered as dead and were sacrificed. Immunohistochemical detection of Ki67 (anti-Ki67 SP6 antibody, Abcam, #ab16667) in tumor tissues were detected as described in the previous methods at fourteen days after immunization. In all experiments, treated groups were randomized to prevent cage effects.

### Statistical analyses

For parametric data in each experiment, the difference between the two groups was statistically analyzed with unpaired two-tailed Student's *t*-tests. Survival curves were analyzed by a log-rank test. All statistical analyses were performed with Graph-Pad Prism software. Increasing levels of confidence are displayed as *P ≤ 0.05, **P ≤ 0.01, ***P ≤ 0.001. All of these data are acquired with four repeated experiments.
